# Supplementary material for: Correlation between Executive Network Integrity and Sarcopenia in Patients with Parkinson’s Disease
Source: Int J Environ Res Public Health. 2019 Dec 4;16(24):4884. doi: 10.3390/ijerph16244884 (PMC6950743; doi:10.3390/ijerph16244884)
Supplement: Supplementary file 1 [file ijerph-16-04884-s001.pdf]

DTI Data Processing

The magnetic resonance images were preprocessed using the FSL v5.0.4 (Functional Magnetic Resonance Imaging of the Brain (FMRIB) Software Library, Oxford, UK; http://www.fmrib.ox.au.uk/fsl), including eddy current correction and brain tissue extraction [1]. Each diffusion-weighted image was registered to the nondiffusion-weighted image by an affine registration approach that was supplied in FMRIB's Linear Image Registration Tool (FLIRT; part of FSL), which was used not only to minimize any image distortion from eddy currents induced by the fast-switching of the gradient coil, but also to reduce any image distortion due to simple head motion. The images were then skull-stripped to remove nonbrain tissue and background noise using the Brain Extraction Tool (BET; part of FSL). A diffusion tensor model was fitted in each voxel using FMRIB's Diffusion Toolbox (FDT, part of FSL) for the calculation of FA values; axial diffusivity (AD) values, $\lambda 1$; radial diffusivity (RD) values, $(\lambda 2+\lambda 3)/2$; and mean diffusivity (MD) values, $(\lambda 1+\lambda 2+\lambda 3)/3$.

The Montreal Neurological Institute (MNI) space served as the standard template space for group comparisons in this study. The FMRIB58_FA standard space image, a high-resolution average of 58 well-aligned good quality FA images in FSL, was used as the target image. Each subject's FA image was spatially normalized to the target image with a nonlinear registration using FMRIB's Non-Linear Image Registration Tool (FNIRT, part of FSL). The final voxel size of an image was resampled to 1 mm cubic resolution. All normalized FA images were smoothed with an 8-mm Gaussian kernel.

References

1. Jenkinson M, Beckmann CF, Behrens TE, Woolrich MW, Smith SM. FSL. *Neuroimage.* 2012;62(2):782-90. doi:10.1016/j.neuroimage.2011.09.015
